# Major Discrimination, Race-Ethnicity, and Inflammation Among Older Adults

**DOI:** 10.1093/geroni/igab046.1326

**Published:** 2021-12-17

**Authors:** Ryon Cobb, Lauren Parker, Roland Thorpe, Jr.

**Affiliations:** 1 University of Georgia, Athens, Georgia, United States; 2 Johns Hopkins Bloomberg School of Public Health, Baltimore, Maryland, United States

## Abstract

This study examines the relationship between self-reported instances of major discrimination and inflammation among older adults, and explores whether this relationship varies in accordance with race/ethnicity. Data from 2006/2008 Health and Retirement Study was used to collect measures of self-reported instances of major discrimination and high-risk C-reactive protein (CRP), which was assayed from blood samples. Modified Poisson regression with robust standard errors was applied to estimate the prevalence ratios of self-reported instances of major discrimination, as it relates to high-risk CRP (CRP ≥ 22 kg/m2), and test whether this relationship varies by race/ethnicity. Respondents who experienced any instances of major discrimination had a higher likelihood of high-risk CRP (prevalence ratio [PR]: 1.14, 95% confidence interval [CI] = 1.07–1.22) than those who did not report experiencing any instances of major discrimination. This relationship was weaker for blacks than whites (PR: 0.81, 95% CI = 0.69–0.95).

